# Fluorination‐Enhanced Ambient Stability and Electronic Tolerance of Black Phosphorus Quantum Dots

**DOI:** 10.1002/advs.201800420

**Published:** 2018-06-13

**Authors:** Xian Tang, Hong Chen, Joice Sophia Ponraj, Sathish Chander Dhanabalan, Quanlan Xiao, Dianyuan Fan, Han Zhang

**Affiliations:** ^1^ Shenzhen Engineering Laboratory of Phosphorene and Optoelectronics Collaborative Innovation Center for Optoelectronic Science and Technology, and Key Laboratory of Optoelectronic Devices and Systems of Ministry of Education and Guangdong Province College of Optoelectronic Engineering Shenzhen University Shenzhen 518060 China; ^2^ School of Materials Science and Energy Engineering Foshan University Foshan 528000 China; ^3^ Department of Nanoscience and Technology Bharathiar University Coimbatore 641046 India

**Keywords:** defect tolerance, density functional theory (DFT) calculations, electrochemical exfoliation, fluorinated phosphorene quantum dots, trap states

## Abstract

The environmental instability and uneliminable electronic trap states in black phosphorus quantum dots (BPQDs) limit the optoelectronics and related applications of BPQDs. Here, fluorinated BPQDs (F‐BPQDs) are successfully synthesized by using a facile electrochemical exfoliation and synchronous fluorination method. The F‐BPQDs exhibit robust ambient stability and limited fluorination capability, showing a nonstoichiometric fluorination degree (*D*_F_) maximum of ≈0.68. Density functional theory calculations confirm that due to the edge etching effect of fluorine adatoms, the simulated F‐BPQDs become structurally unstable when *D*_F_ surpasses the limit. Furthermore, the trap states of BPQDs can be effectively eliminated via fluorination to obtain a coordination number of 3 or 5 for fluorinated and unfluorinated phosphorus atoms. The results reveal that the air‐stable F‐BPQDs exhibit fluorine defect‐enhanced electronic tolerance, which is crucial for nanophotonics and nanoelectronics applications.

## 1 Introduction

Quantum dots (QDs) have been attracting increasing interest owing to their small size‐induced quantum confinement and edge effects.^1^ QDs derived from 2D materials, including graphene,^2^ graphic‐C_3_N_4_,^3^ MoS_2_,^4^ and phosphorene,^5^ display several advantages, such as photostability and low toxicity in par with the conventional organic fluorophores and semiconductor QDs.^6^ Given their novel optical and electronic properties, 2D material‐derived QDs have widespread applications in bioimaging,^7^ photodynamic therapy,^8^ photo‐/electrocatalysis,^9^ photodetection,^10^ and sensing.^11^ Black phosphorus QDs (BPQDs) or phosphorene QDs possess unique features, such as broadband absorption^5^, ^13^ and photoluminescence in the violet–green^14^ and near‐infrared (NIR)^15^ regions, owing to the special washboard‐like layered structure combined with the tunable bandgap (0.3–2.0 eV) of phosphorene.^12^ However, BPQDs are subjected to environmental degradation, similar to that of phosphorene.^16^ This issue severely hinders the efficiency and controllability of BPQDs when employed for cancer treatment,^17^, ^18^ energy devices,^19^ and ultrafast photonics.^20^ Consequently, a stabilization strategy is urgently required for BPQDs.

Few methods have been reported for protecting phosphorene from ambient oxidation and degradation; these methods mainly include covalent and noncovalent functionalization^21^ and shell encapsulation.^22^ The robust air‐stability of phosphorene has been achieved. However, the stability issue of BPQDs has received less attention. Our previous studies suggested that BPQDs functionalized by polyethylene glycol[[qv: 5b]] or poly(lactic‐*co*‐glycolic acid)^17^ exhibit controllable degradation rate, which is beneficial for BPQD‐based photothermal therapy. Given the protection supplemented by polymethyl methacrylate (PMMA), electrospun BPQD/PMMA nanofiber films show stable nonlinear saturable absorption up to 3 months.^20^ We have recently developed an electrochemical exfoliation and synchronous fluorination (EESF) approach to deliver a novel phosphorene derivative, fluorinated phosphorene (FP), which possesses superior air stability due to its fluorine adatom‐induced antioxidation and antihydration behavior.^23^ In contrast to previous stabilization methods for phosphorene^21^, ^22^ and BPQDs,^17^, ^20^ this facile and one‐step strategy does not need any post‐treatment or step‐by‐step synthesis of functional groups. Fluorinated BPQDs (F‐BPQDs) are expected to be produced in association with the exfoliated FP because of the one‐pot synthesis feature of ionic‐liquid‐assisted electrochemical exfoliation.^24^

Semi‐empirical tight‐binding simulations have shown that BPQDs possess size‐, defect‐, and external field‐controlled edge trap states in the energy gap regardless of their shape and edge morphology.^25^ Trap states assist carrier recombination and block electronic interlevel transitions, and thus exert profound effects on the optoelectronics of QDs.^26^ However, recent density functional theory (DFT) studies have revealed that defective QDs have crucial electronic tolerance to their structural defects, and the trap states of defective QDs can be effectively modulated to preserve their essential electronic and optical properties.^27^ In particular, the trap states of defective BPQDs can be eliminated depending on the coordination number (CN) of P atoms,^28^ offering a broad space to fine‐tune the superior optoelectronics and clarify the luminescence mechanisms of BPQDs. Consequently, exploring the electronic tolerance of BPQDs and modulating their trap states through fluorination are important. In this contribution, F‐BPQDs are fabricated by using the as‐proposed EESF method at different electrolyte concentrations (*C*_E_). The ambient stability of F‐BPQDs is also revealed. The effect of fluorination degree (*D*_F_) on the structural and electronic properties of F‐BPQDs is investigated by DFT.

## 2 Results and Discussion

### 2.1 Synthesis and Ambient Stability of F‐BPQDs

**Figure 1:**
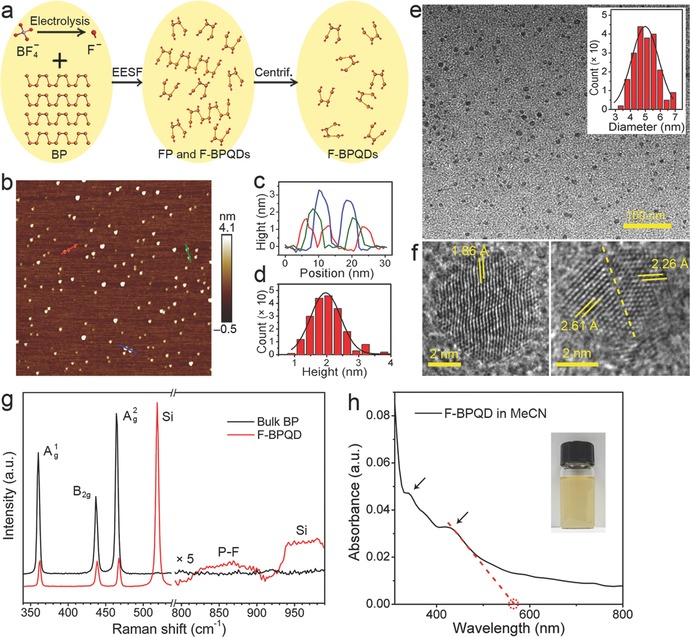
Synthesis and characterizations of F‐BPQDs. a) Schematic of the synthesis of F‐BPQDs. b) AFM image of F‐BPQDs. c) Height profiles along the red, blue, and green lines in (b). d) Statistical histogram of the heights of F‐BPQDs measured from (b). e) TEM and f) HRTEM images of F‐BPQDs. The inset in (e) provides the diameter distribution histogram of the acquired F‐BPQDs. The dashed line in (f) indicates the lattice dislocation in the F‐BPQD. g) Raman spectra of F‐BPQDs and bulk BP. The spectra in the region of 800–990 cm^−1^ are multiplied by a scaling factor of 5. h) UV–vis–NIR absorption spectrum of F‐BPQD/MeCN dispersion. The arrows mark the absorption shoulders. The red dashed lines show a linear fit of the band edge of the first absorption peak. The inset is a photograph of the dispersion.

Synthesis of F‐BPQDs is schematically shown in **Figure** **1**a. During the EESF, the 1‐ethyl‐3‐methylimidazolium tetrafluoroborate ([EMIM][BF_4_])/acetonitrile (MeCN) electrolyte used was discolored from transparent to light yellow because of the generation of bulk BP exfoliates.^29^ However, the standing electrolyte persisted to develop a deep yellow color after EESF, suggesting that the color reaction of MeCN with F^−^ occurred in the electrolyte^30^

(1) $$2F^{-}\;+\;{\mathrm{CH}}_{3}\mathrm{CN}\to {\mathrm{HF}}_{2}^{-}+{\mathrm{CH}}_{2}{\mathrm{CN}}^{-}$$

Therefore, the electrolysis of ${\mathrm{BF}}_{4}^{-}$ to F^−^ in EESF can be deduced according to the following reaction^31^

(2) $${\mathrm{BF}}_{4}^{-}\to {\mathrm{BF}}_{3}+F^{-}$$

where the BF_3_ molecules can also be trapped by MeCN to form donor–acceptor bonds,^32^ and the reaction was expected to be activated by the applied anodic voltage of +8 V versus Ag wire. Therefore, the one‐pot exfoliates of BP were synchronously fluorinated by ${\mathrm{BF}}_{4}^{-}$‐electrolyzed F^−^ anions. The F‐BPQDs were then separated from the fluorinated exfoliates by centrifugation at 15 000 rpm.

The atomic force microscopy (AFM) image of the F‐BPQDs synthesized by the 0.1 m [EMIM][BF_4_]/MeCN electrolyte is shown in Figure 1b. The F‐BPQDs show high selectivity and uniform morphology. No agglomeration of the nanodots is observed, suggesting good dispersion in the solvent. The height profiles of several F‐BPQDs (plotted in Figure 1c) demonstrate that the thickness ranges from 1.2 to 3.5 nm, corresponding to 2–7 atomic layers. Statistical AFM analysis (Figure 1d) indicates that the heights of 150 counted F‐BPQDs exhibit a Guassian distribution with an average thickness of 2.0 ± 1.2 nm. The transmission electron microscopy (TEM) image shown in Figure 1e reveals that the F‐BPQDs have a relatively spherical shape with an average diameter of 5.0 ± 2.0 nm (see the diameter distribution histogram of 150 counted F‐BPQDs in the inset of Figure 1e). High‐resolution TEM (HRTEM) images of two individual F‐BPQDs nanodots are shown in Figure 1f. The figure shows that one F‐BPQD (left panel) has a single crystalline feature but with slightly distorted atomistic texture, which is probably generated in the fluorination processes. The other (right panel) figure shows evident lattice dislocation as indicated by the white dashed line. The measured lattice distances of the nanocrystals are 1.86, 2.61, and 2.26 Å, which are close to the (121), (004), and (014) interplane distances of bulk BP, respectively.^33^

The Raman spectrum of the F‐BPQDs is shown in Figure 1g. The phosphorous $A_{g}^{1}$, *B*_2g_, and $A_{g}^{2}$ phonon modes located at 362.5, 438.7, and 467.5 cm^−1^, respectively, red‐shifted as compared with the bulk BP, which is similar to that observed in FP.^23^ However, these modes were significantly suppressed by the fluorination‐induced structural changes, similar to the case of fluorinated graphene.^34^ In addition, a weak broad band ranging from 800 to 910 cm^−1^ appeared and peaked at ≈868 cm^−1^. This peak has been assigned to the F–P–F stretching vibrations in fluorophosphate glasses^35^ and PCl_2_F_3_ molecules.^36^ Thus, similar PF_2_‐containing configurations can be deduced in the F‐BPQDs. As *C*_E_ increased, the synthesized F‐BPQDs exhibited increased Raman vibration suppression (Figure S1, Supporting Information), suggesting increased *D*_F_. The optical absorption spectrum of F‐BPQD/MeCN dispersion is shown in Figure 1h. In contrast to the pure BPQDs that exhibited smoothly increasing absorption from the NIR to UV region,[[qv: 5b,17]] F‐BPQDs demonstrated distinct absorption shoulders at 430 and 340 nm. Similar peaks have been previously predicted by DFT calculations on the absorption spectra of pure BPQDs, which exhibited two prominent visible–UV peaks ascribed to the electron excitation transitions from the optimized ground state to the first excited singlet state.[[qv: 28a]] The two peaks were different from the layer‐dependent absorption peaks of the few‐layer phosphorene^37^ and FP^23^ at the NIR region. The white dashed line in Figure 1h yielded a band edge of the first absorption peak at ≈565 nm.

**Figure 2:**
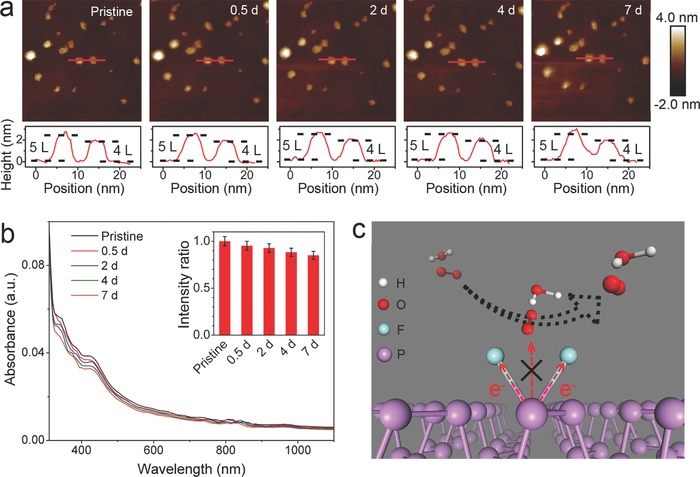
Ambient stability of F‐BPQDs. a) AFM images showing evolution of the morphology of pristine F‐BPQDs under persistent exposure to ambient conditions for 7 d. b) Time‐dependent absorption spectra of pristine F‐BPQDs dispersion for 7 d, with the inset histogram showing the variation of absorbance intensity at 425 nm. c) Schematic model illustrating the stabilization mechanism of F‐BPQDs in air.

The environmental stability of F‐BPQDs was further evaluated because the instability of BPQDs and phosphorene is a serious issue hindering their practical applications. Time‐dependent AFM images of the F‐BPQDs synthesized in 0.1 m [EMIM][BF_4_]/MeCN electrolyte are shown in **Figure** **2**a. During the persistent ambient exposure for 0.5, 2, 4, and 7 d, the pristine F‐BPQDs dispersed on the Si substrate exhibited no significant degradation features, such as bubbling and depletion of atomic layers, which had been observed in BPQDs and phosphorene.^16^, ^17^ The height profiles of the two recorded F‐BPQDs confirmed the morphological integrity during the exposure period. Therefore, the resulting F‐BPQDs exhibit robust environmental stability. The robust ambient stability was also confirmed by the absorption spectra of the F‐BPQD dispersion (Figure 2b), which showed that the absorbance decreased slightly during the 7 d of standing. As shown in the inset of Figure 2b, the absorbance intensity at 425 nm decreased by 13.8%, which did not significantly change the optics of the F‐BPQDs. By contrast, the time‐dependent AFM images of the control sample BPQDs (Figure S2 in Supporting Information) show that the BPQDs degraded rapidly and eventually disappeared during the ambient exposure for 12 h. The stabilization mechanism of F‐BPQDs in air is illustrated in Figure 2c. Our DFT calculations^23^ on the Bader charge distribution of FP showed that the highly electronegative fluorine adatoms attracted electrons from the P atoms and formed a strong P—F bond. When the O_2_ and H_2_O molecules approached the F‐BPQD surface, no significant charge was found in the fluorinated P atoms to transfer to the O atoms, i.e., the charge transfer pathway between the P and O atoms was blocked. As a result, the general degradation processes of phosphorene,^16^ including oxidation and subsequent formation of phosphoric and phosphorus acids, did not occur. Subsequently, O_2_ and H_2_O were not decomposed and were repelled from the F‐BPQD surface, endowing F‐BPQDs with antioxidation and antihydration properties.

### 2.2 Effect of *D*_F_ on the Structural and Electronic Properties of F‐BPQDs

**Figure 3:**
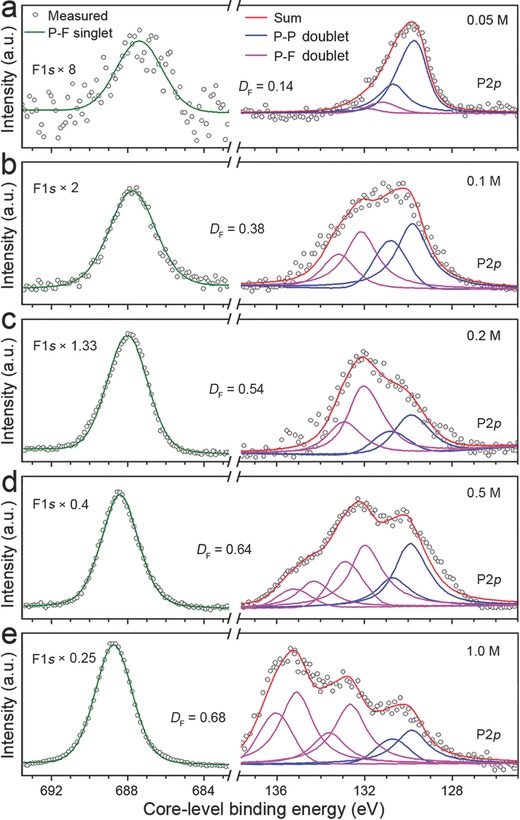
F 1s and P 2p XPS spectra of F‐BPQDs synthesized by using a) 0.05 m, b) 0.1 m, c) 0.2 m, d) 0.5 m, and e) 1.0 m [EMIM][BF_4_]/MeCN electrolyte. The F 1s spectra are multiplied by the indicated scaling factors. The F 1s spectra are deconvoluted with a singlet. The P 2p spectra are deconvoluted with two or three P 2p_3/2_ and 2p_1/2_ doublets belonging to the unfluorinated (P–P) and fluorinated (P–F) parts of F‐BPQDs. *D*_F_ of each sample is also presented.

*D*_F_ is a key factor that determines the properties of fluorides. Herein, *D*_F_ (or fluorine concentration) is defined as the atomic F/P ratio in QDs. X‐ray photoelectron spectroscopy (XPS), a feasible approach for quantitative composition analysis, has been used to determine the *D*_F_ of fluorinated graphene.^38^ The F 1s and P 2p XPS spectra of the F‐BPQDs synthesized in 0.05, 0.1, 0.2, 0.5, and 1.0 m [EMIM][BF_4_]/MeCN electrolyte are shown in **Figure** **3**. The peak information in the XPS spectra is summarized in Table S1 (Supporting Information). In general, Figure 3a–e shows that the F 1s peak intensity increased as *C*_E_ increased, suggesting that the P—F bond formation and *D*_F_ of the F‐BPQDs increased. The F 1s peak was shifted to higher binding energies as *C*_E_ increased, from 687.5 eV for 0.05 m to 688.5 eV for 1.0 m, indicating the transformation of PF*_x_* (0 < *x* ≤ 3) species to those with larger *x* values for the F‐BPQDs,^39^ especially to PF_2_ and PF_3_. The P 2p XPS spectra of the F‐BPQDs were consistent with the P 2p_3/2_ and 2p_1/2_ doublets belonging to the unfluorinated and fluorinated parts of F‐BPQDs. In general, mono‐, bi‐, and trifluorinated P sites are present in the surfaces and edges of an F‐BPQD according to the degree of bond saturation of P atoms, as schematically shown in Figure S3 (Supporting Information). At *C*_E_ = 0.05 m, the P 2p spectrum was deconvoluted with a small binding‐energy fluorinated doublet located at 131.0 and 131.9 eV and a large unfluorinated doublet at 129.9 and 130.6 eV. Thus, the F‐BPQDs may have a small portion of monofluorinated P sites in the surfaces and armchair edges, considering the DFT‐calculated core‐level binding energy shifts of FP^23^ and previous XPS measurements on PF‐containing species.^40^ The portion and binding energy position of the fluorinated doublet increased and shifted to 132.0–133.1 eV as *C*_E_ increased to 0.1 and 0.2 m, indicating the increased *D*_F_ as well as the formation of monofluorinated P sites in the zigzag edges and bi‐fluorinated P sites on the surfaces and armchair edges.[[qv: 23,39b,40]] The bifluorinated P sites may be transformed from the monofluorinated P sites by adsorption of another F atom. Another fluorinated P 2p doublet appeared at higher binding energies of 134.4 to 136.0 eV for the F‐BPQDs synthesized at *C*_E_ = 0.5 and 1.0 m, which corresponded to the bifluorinated P sites in the zigzag edges and tri‐fluorinated P sites in the zigzag and armchair edges.^23^ The lower fluorinated doublet was weakened, suggesting the transformation of mono‐ and bifluorinated sites to higher fluorinated sites. These results reveal the important modulation effect of *C*_E_ on *D*_F_ and the atomistic fluorination configurations of the synthesized F‐BPQDs.

The P 2p spectra merely demonstrated a qualitative variation trend of *D*_F_ for the F‐BPQDs synthesized at different *C*_E_ values because the excitation probability of the core electrons of the fluorinated and unfluorinated P atoms is different, and the area ratios of the fitted fluorinated and unfluorinated peaks have large deviations with *D*_F_. As shown in Figure 3a–e, the *D*_F_ values were 0.14, 0.38, 0.54, 0.64, and 0.68 for *C*_E_ = 0.05, 0.1, 0.2, 0.5, and 1.0 m, respectively. *D*_F_ increased rapidly at small *C*_E_ values because abundant unfluorinated sites of the electrochemically exfoliated BPQDs were present for fluorination. *D*_F_ slightly increased as *C*_E_ was increased and, eventually, reached the maximal fluorination capability at 1.0 m. The nominal bond‐saturated BPQDs (with each surficial P atom bonded with two F atoms and each edge atom with three F atoms) would reach a maximal nonstoichiometric *D*_F_ of >2.0; thus, the measured maximal *D*_F_ of 0.68 suggested a small fluorination capability of the F‐BPQDs.

**Figure 4:**
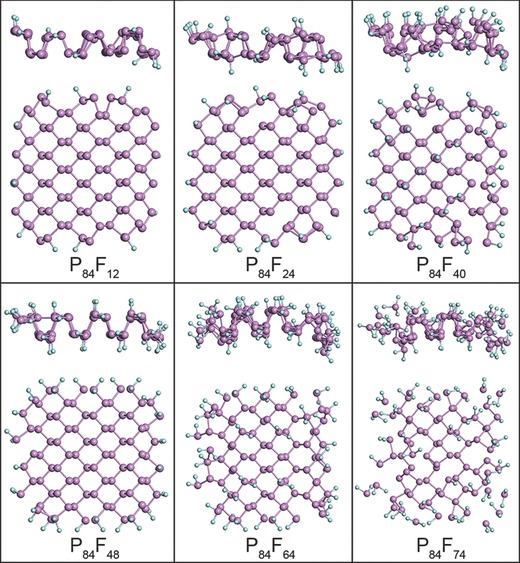
Side and top views of optimized F‐BPQD structures of P_84_F_12_, P_84_F_24_, P_84_F_40_, P_84_F_48_, P_84_F_64_, and P_84_F_74_.

We further calculated the structural stability of F‐BPQDs with different *D*_F_s to explain the unexpected fluorination capability of the F‐BPQDs. The primitive F‐BPQD models were named as P_84_F*_x_*, where *x* is the number of F adatoms. The adsorption characteristics of F adatoms on F‐BPQDs were initially investigated by DFT, and the results are shown in Figures S4 and S5 (Supporting Information). According to the adsorption rules illustrated in Figures S4 and S5 (Supporting Information), five F‐BPQD atomic structures, i.e., P_84_F_12_, P_84_F_24_, P_84_F_40_, P_84_F_48_, P_84_F_64_, and P_84_F_74_ with *D*_F_ = 0.14, 0.29, 0.48, 0.57, 0.76, and 0.88, respectively, were selected and fully optimized. As shown in **Figure** **4**, for P_84_F_12_ with half of the edge atoms being monofluorinated, the relaxed configuration showed slight deformation, and the F atoms were firmly bonded. When the edge atoms were fully monofluorinated, the structural deformation of P_84_F_24_ became evident and a weak P—P bond was broken in the edge corner. However, the structural integrity was well preserved. The P_84_F_40_ F‐BPQD monofluorinated in the edge and surface exhibited severe deformation. Notably, each surface mono‐fluorination site caused P—P bond breaking to maintain the CN of 3, which conferred the porous feature of P_84_F_40_. However, excess surface monofluorination sites may cause segmentation of the F‐BPQDs, due to the theoretically predicted chemical scissor effect.^41^ The relaxed P_84_F_48_ with fully bifluorinated edge sites exhibited dramatic edge evolutions. All the P—P bonds in the armchair edge were broken to maintain a CN of 3, whereas the zigzag edge bifluorination sites seemed to be more stable and exhibited comparatively less bond breakage. When the surface was also bifluorinated to reach a *D*_F_ of 0.57, for P_84_F_64_, the entire phosphorus network was evidently distorted. From the side view, P_84_F_64_ was bent against the fluorinated surface as a result of the drag of the superficial electronegative F adatoms, explaining the nearly spherical shape of the F‐BPQDs. However, contrary to the monofluorination sites that easily caused bond cleavage, the surface bifluorination sites hardly caused bond breakage and maintained the sites with a CN of 5. When the number of surface bifluorination sites increased, the assumed P_84_F_74_ F‐BPQD became unstable. In addition to the severe damage of the structure, several bifluorinated edge P atoms departed from the network to decrease *D*_F_, forming individual PF_2_ radicals. However, no PF_2_ was formed from the surface bi‐fluorination sites. This result suggests that the fluorination‐induced decomposition of F‐BPQDs occurred in the edge etching process to gradually reduce their lateral size. The surface bifluorination sites had a proportion of 0.72 in the surface sites (13 in 18), suggesting that only partial surface bifluorination was allowed for the F‐BPQDs. Evidently, the edge collapse of P_84_F_74_ resulted from the combined effect of edge and surface fluorination, whereas the minority surface fluorination had a profound contribution. The results confirmed that the F‐BPQDs had a relatively small fluorination capability with a theoretical *D*_F_ limit of ≈0.76. The predicted *D*_F_ limit was slightly larger than the experimental value of 0.68 because the synthesized F‐BPQDs had a large size with a small portion of edge F adatoms that can be fully bifluorinated. The structural decomposition became increasingly significant as *D*_F_ was increased. The results of P_84_F_72_, P_84_F_108_, P_84_F_140_, P_84_F_168_, and P_84_F_192_ are shown in Figure S6 (Supporting Information). The nominal bond‐saturated P_84_F_192_ was relaxed to separate into small radicals and molecular chains, including PF_2_, PF_3_, P_2_F_4_, P_2_F_5_, P_3_F_7_, and P_4_F_12_. Moreover, the F‐BPQDs with fully tri‐fluorinated edge sites (P_84_F_72_) were thermodynamically unstable; the zigzag edge was stable, but the armchair edge was etched to form PF_3_ species (Figure S6, Supporting Information).

**Figure 5:**
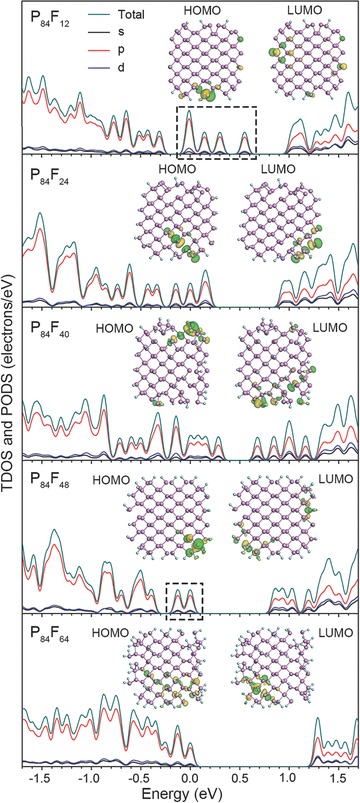
TDOS and s‐, p‐, and d‐orbital projected PDOS of P_84_F_12_, P_84_F_24_, P_84_F_40_, P_84_F_48_, and P_84_F_64_. The dashed rectangles denote trap states in P_84_F_12_ and P_84_F_48_. Wave‐function isosurfaces of HOMO and LUMO in each F‐BPQD are shown in the inset of each panel. *E*_F_ is set to 0 eV.

The dependence of the electronic properties of F‐BPQDs on *D*_F_ was further explored. The DFT‐calculated electronic total density of states (TDOS) and orbital‐projected partial DOS (PDOS) of the optimized P_84_F_12_, P_84_F_24_, P_84_F_40_, P_84_F_48_, and P_84_F_64_ are shown in **Figure** **5**. The energy spectra of these F‐BPQDs are shown in Figure S7 (Supporting Information). From TDOS and PDOS of Figure 5, the highest occupied molecular orbital (HOMO) and the lowest unoccupied molecular orbital (LUMO) of these F‐BPQDs near the Fermi level (*E*_F_, set to zero) were mainly derived from the p orbitals of the P or F atoms, whereas the 3s and 3d orbitals of the P atoms had minor contributions. The sp^3^d hybridization‐induced 3s–3d electron transitions were confirmed. For P_84_F_12_, four evident TDOS peaks (denoted by the dashed rectangle) appeared in the electronic gap; these peaks were well separated from the nearby collective states and belonged to the deep trap states. From the energy spectrum of P_84_F_12_ (Figure S7, Supporting Information), the trap states consisted of HOMO‐1, HOMO, LUMO, LUMO+1, and LUMO+2 energy levels. As shown in the inset of the DOS panel of P_84_F_12_, the HOMO and LUMO wave functions were mainly concentrated in the unfluorinated edge P atoms with CN = 2, suggesting that these trap states were induced by edge bonding configurations but not by fluorination. The deep edge trap states, which were essentially localized, would have profound influence on the photoabsorption and emission of F‐BPQDs. For P_84_F_24_ with each edge P atom bonded with an F atom to have a CN of 3, the trap states were completely eliminated from the energy gap, indicating the absence of electron trapping near *E*_F_. The HOMO and LUMO charge‐density regions migrated to the fluorinated edge P atoms as well as to the interior region of the F‐BPQD. Therefore, F‐BPQDs possess electronic tolerance to fluorination‐induced defects. The case of P_84_F_40_ was similar to that of P_84_F_24_, given that the monofluorinated edge and surface P atoms of P_84_F_40_ had a CN of 3. However, relatively shallow trap states appeared for P_84_F_48_ with each edge P atom bifluorinated to obtain a CN of 4, and the HOMO and LUMO charge‐density regions returned to the edge. The trap states were eliminated again in the even more defective P_84_F_64_. In‐depth structural analysis showed that each bi‐fluorinated edge and surface P atom in P_84_F_64_ had a CN of 3 and 5, respectively. Thus, in addition to the CN = 3 rule,^28^ the F‐BPQDs with CN = 5 also had the defect‐tolerance to exclude trap states. The energy spectrum of P_84_F_64_ (Figure S7, Supporting Information) provided a HOMO‐LUMO gap of 1.28 eV, in accordance with the NIR absorption of F‐BPQDs shown in Figures 1h and 2b. The defect‐tolerance of P_84_F_24_, P_84_F_40_, and P_84_F_64_ with CN = 3 or 5 for P atoms may be ascribed to the overall electronic stoichiometry of the F‐BPQDs as observed in other QDs.[[qv: 27b]] The calculated results suggest that the trap states of F‐BPQDs can be effectively eliminated by *D*_F_ for electronic and optoelectronic applications.

## 3 Conclusion

Highly selective F‐BPQDs with an average size of 5.0 ± 2.0 nm and an average thickness of 2.0 ± 1.2 nm were synthesized by using a one‐step EESF method. The F‐BPQDs were experimentally found to possess robust ambient‐stability owing to the fluorine adatom‐induced blocking of the charge transfer pathway from the P atoms to the external oxygen. The *D*_F_ of the F‐BPQDs increased with the increase in *C*_E_. In particular, the F‐BPQDs may be subjected to etching of fluorine adatoms and thus exhibited limited fluorination capability of reaching a maximal *D*_F_ of ≈0.68. DFT calculations confirmed that as *D*_F_ increased, the assumed F‐BPQDs were subjected to microstructural deformation, edge collapse, and eventually decomposition to small PF species. Furthermore, the trapping states of F‐BPQDs can be modulated by fluorine defects‐controlled CN of the edge and surface P atoms, for which a CN of 3 or 5 is a prerequisite to eliminate the trapping states and preserve the electronic tolerance. The results reveal that fluorination is an effective pathway not only for enhancing the environmental stability but also for modulating the electron transition‐related properties of BPQDs.

## 4 Experimental Section

*Synthesis of F‐BPQDs*: F‐BPQDs were synthesized according to an EESF method.^23^ An intact BP chunk (99.998%, Smart Elements) was used as the working electrode in a sealed three‐electrode electrochemical cell containing [EMIM][BF_4_] (98.0%, Aladdin, 0.05, 0.1, 0.2, 0.5, or 1.0 m) in MeCN (gradient grade, ≥99.9%, LiChrosolv, 150 mL) as the electrolyte. A Pt wire and an Ag wire were used as the counter electrode and the quasireference electrode, respectively. By applying a constant potential of +8 V versus Ag wire to the working electrode, the BP chunk was electrochemically exfoliated and synchronously fluorinated by the electrolyzed anions. The exfoliates/electrolyte dispersions obtained were centrifuged at 15 000 rpm for 40 min to separate the F‐BPQDs from the exfoliates. The control sample BPQDs was synthesized by sonication.[[qv: 5b]]

*Characterizations*: Height and morphology of the synthesized F‐BPQDs and BPQDs were observed with the aid of an ICON Bruker AFM system in tapping mode at a scan rate of 1.79 Hz. TEM and HRTEM images of the F‐BPQDs were acquired from the FEI Tecnai G^2^ F30 field‐emission TEM equipment operated at an accelerating voltage of 300 kV. Raman spectra of the F‐BPQDs and bulk BP were acquired by Renishaw inVia confocal Raman microscope equipped with a 514 nm Ar ion laser as the excitation source. UV–vis–NIR spectra of the F‐BPQD dispersion were acquired using a Perkin‐Elmer LAMBDA 750 Spectrophotometer in an incident light wavelength range of 200 to 2000 nm. XPS was performed on a ULVAC PHI 5000 VersaProbe II using an Al Kα (λ = 0.83 nm, *h*_υ_ = 1486.7 eV) X‐ray source operated at 23.5 W. *D*_F_ was calculated as *D*_F_ = *I*_1_ *S*_2_/*I*_2_ *S*_1_, where *I*_1_ and *S*_1_ were the area and sensitivity factor of F 1s peak, and *I*_2_ and *S*_2_ were those of P 2p peak.

*DFT Calculations*: The all‐electron DFT calculations were performed with the DMol^3^ program in the Materials Studio 8.0 (BIOVIA) suite.^42^ The Perdew–Burke–Ernzerhof exchange‐correlation functional at the generalized gradient approximation level^43^ was used and formed by a double numerical basis set including polarization functions (DNP 4.4) and Tkatchenko‐Scheffler corrections. The convergence criteria for geometry optimizations were 10^−5^ Ha, 0.004 Ha Å^−1^, and 0.005 Å for energy, force, and displacement, respectively. All the calculations employed a self‐consistent field (SCF) tolerance of 10^−5^ and an orbital cutoff of 4.2 Å. SCF convergence was accelerated by using a Pulay's direct inversion of iterative subspace with a maximum size of 6, a charge density preconditioner with a reference wave vector of 4.0 a_0_^−1^, and a thermal smearing of 0.01 Ha. DOS was smeared with a width of 0.03 eV. HOMO and LUMO were sampled on a grid with a spacing of 0.2 Å. We calculated the electronic structures of H‐passivated BPQD P_84_H_24_ to confirm the reliability of the exchange‐correlation function used. The results were in good agreement with previous results calculated with the Becke three‐parameter Lee‐Yang‐Parr functional (Figure S8, Supporting Information).^28^

## Conflict of Interest

The authors declare no conflict of interest.

## Supporting information

Supplementary
