# Supplementary material for: Fluorination‐Enhanced Ambient Stability and Electronic Tolerance of Black Phosphorus Quantum Dots
Source: Adv Sci (Weinh). 2018 Jun 13;5(9):1800420. doi: 10.1002/advs.201800420 (PMC6145272; doi:10.1002/advs.201800420)
Supplement: Supplementary file 1 — Supplementary [file ADVS-5-1800420-s001.pdf]

Copyright WILEY-VCH Verlag GmbH & Co. KGaA, 69469 Weinheim, Germany, 2018.

ADVANCED SCIENCE
Open Access

Supporting Information

for *Adv. Sci.,* DOI: 10.1002/advs.201800420

Fluorination-Enhanced Ambient Stability and Electronic Tolerance of Black Phosphorus Quantum Dots

Xian Tang, Hong Chen, Joice Sophia Ponraj, Sathish Chander Dhanabalan, Quanlan Xiao, Dianyuan Fan, and Han Zhang**

Copyright WILEY-VCH Verlag GmbH & Co. KGaA, 69469 Weinheim, Germany, 2016.

Supporting Information

Fluorination-Enhanced Ambient Stability and Electronic Tolerance of Black Phosphorus Quantum Dots

Xian Tang, Hong Chen, Joice Sophia Ponraj, Sathish Chander Dhanabalan, Quanlan Xiao, Dianyuan Fan, and Han Zhang**

Dr. X. Tang, Dr. S. C. Dhanabalan, Dr. Q. Xiao, Prof. D. Fan, Prof. H. Zhang
Shenzhen Engineering Laboratory of Phosphorene and Optoelectronics,
Collaborative Innovation Center for Optoelectronic Science and Technology,
and Key Laboratory of Optoelectronic Devices and Systems of Ministry of Education and Guangdong Province,
College of Optoelectronic Engineering,
Shenzhen University,
Shenzhen 518060, China
E-mail: hzhang@szu.edu.cn

Dr. X. Tang, Prof. H. Chen
School of Materials Science and Energy Engineering, Foshan University, Foshan 528000, China
E-mail: chenghongcs@126.com

Dr. J. S. Ponraj
Department of Nanoscience and Technology,
Bharathiar University, Coimbatore 641046, India

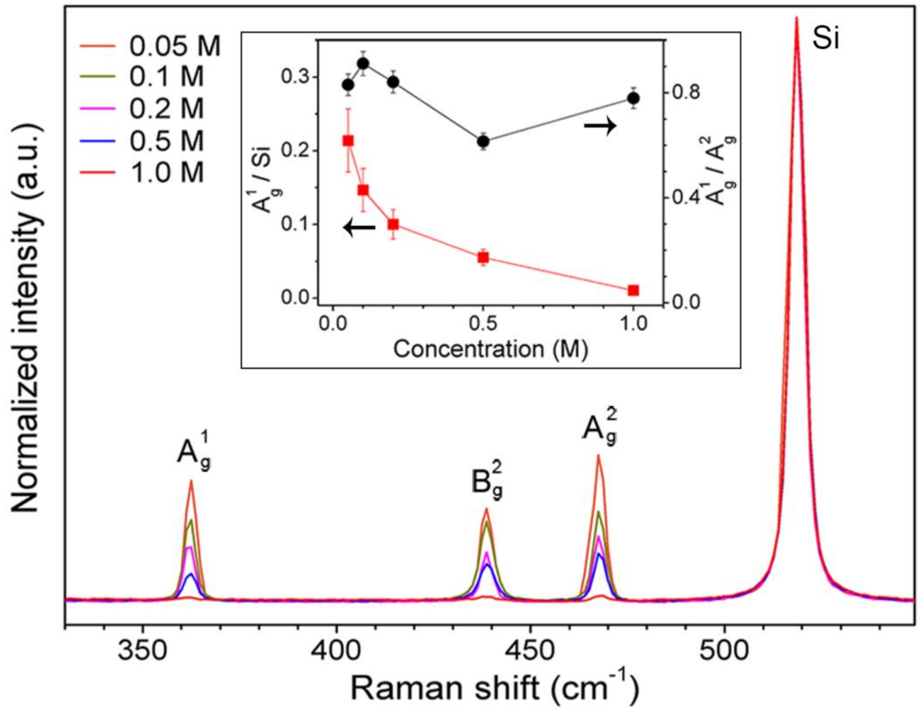

Figure S1. Raman spectra of F-BPQDs synthesized by using [EMIM][BF_4]/MeCN electrolyte at C_E = 0.05, 0.1, 0.2, 0.5, and 1.0 M. The inset shows variations of the peak intensity ratios of A_g^1/Si and A_g^1/A_g^2 with C_E. It can be seen that while the vibration positions of the A_g^1, B_{2g} and A_g^2 modes were not obviously shifted, the vibration strength was significantly decreased with the increase of C_E.

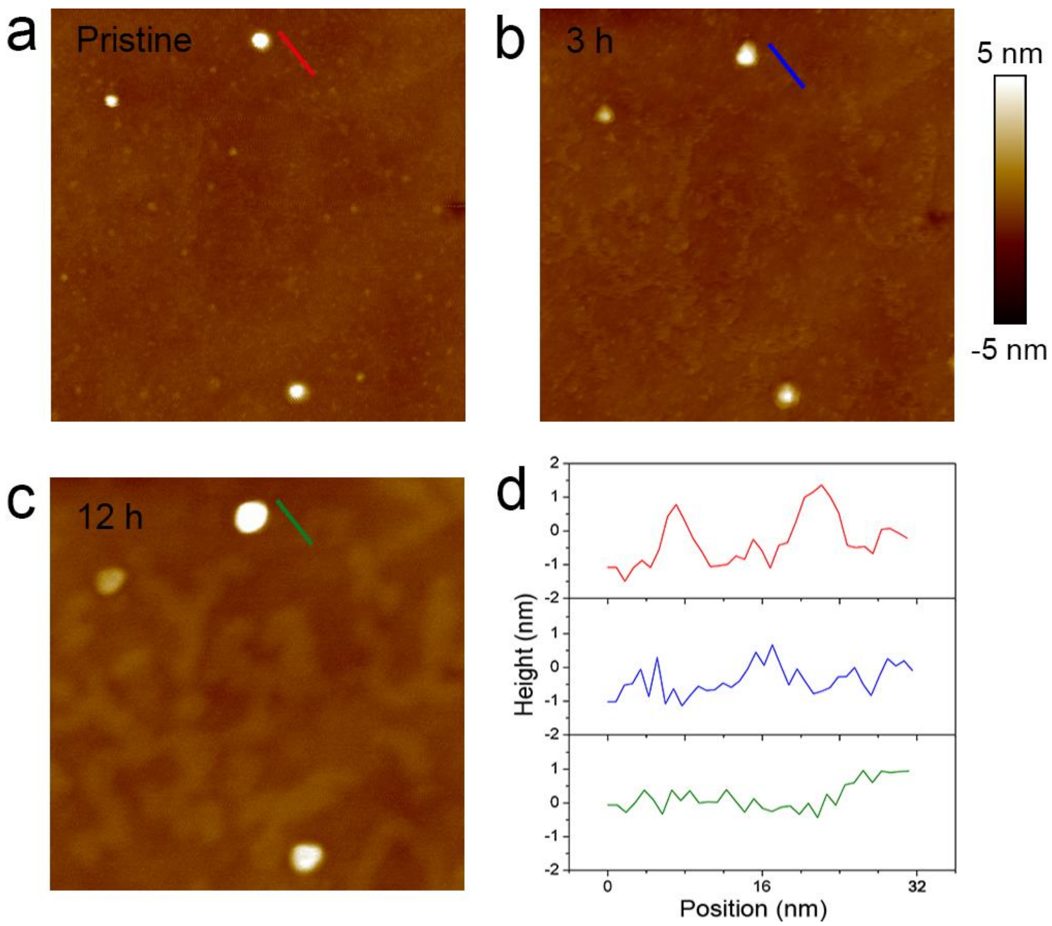

Figure S2. AFM images showing the degradation process of BPQDs under persistent exposure to ambient conditions: (a) pristine; (b) 3 h; (c) 12 h. (d) Height profile evolution of a same position denoted by the solid straight lines in (a)-(c).

Table S1. Information summarization of F 1s and P 2p (with fitted doublets) peaks in the XPS spectra of F-BPQDs synthesized by using 0.05–1.0 M [EMIM][BF_4]/MeCN electrolyte shown in Figure 3.

C_E/M	F 1s		P 2p						D_F
	Position/eV	Area/a.u.	P-P doublet		P-F doublet-1		P-F doublet-2		
			Position/eV	Area/a.u.	Position/eV	Area/a.u.	Position/eV	Area/a.u.	
0.05	687.5	665.2	129.9/ 130.6	1685.5/ 842.7	131.0/ 131.9	214.6/ 107.2	N/A	N/A	0.14
0.1	687.7	6167.7	129.9/ 130.6	3466.5/ 1733.3	132.0/ 133.1	3025.1/ 1512.5	N/A	N/A	0.38
0.2	688.0	8868.1	129.9/ 130.6	2430.5/ 1215.2	132.0/ 133.0	4138.4/ 2069.2	N/A	N/A	0.54
0.5	688.3	12568.9	129.9/ 130.6	3135.4/ 1567.7	132.0/ 133.0	3323.1/ 1661.5	134.4/ 135.3	1360.5/ 680.3	0.64
1.0	688.5	13203.4	129.9/ 130.6	1587.6/ 793.8	132.8/ 133.8	2828.4/ 1414.2	135.0/ 136.0	3290.8/ 1645.4	0.68

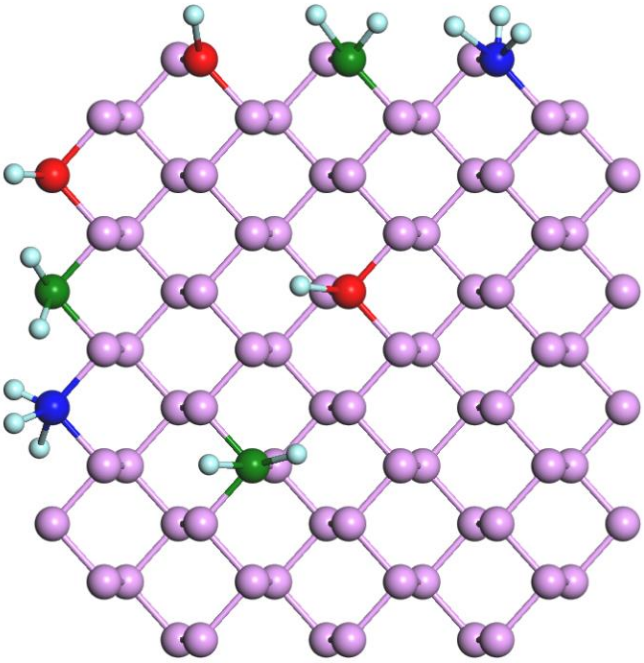

Figure S3. Schematic illustration of possible fluorinated P sites of an F-BPQD or fluorinated phosphorene. Red: mono-fluorinated site in the surface, zigzag edge, and armchair edge. Green: bi-fluorinated in the surface, zigzag edge, and armchair edge. Blue: Tri-fluorinated in zigzag and armchair edges. Note that the surficial tri-fluorinated site is not possible because of the bond saturation of the P atoms.

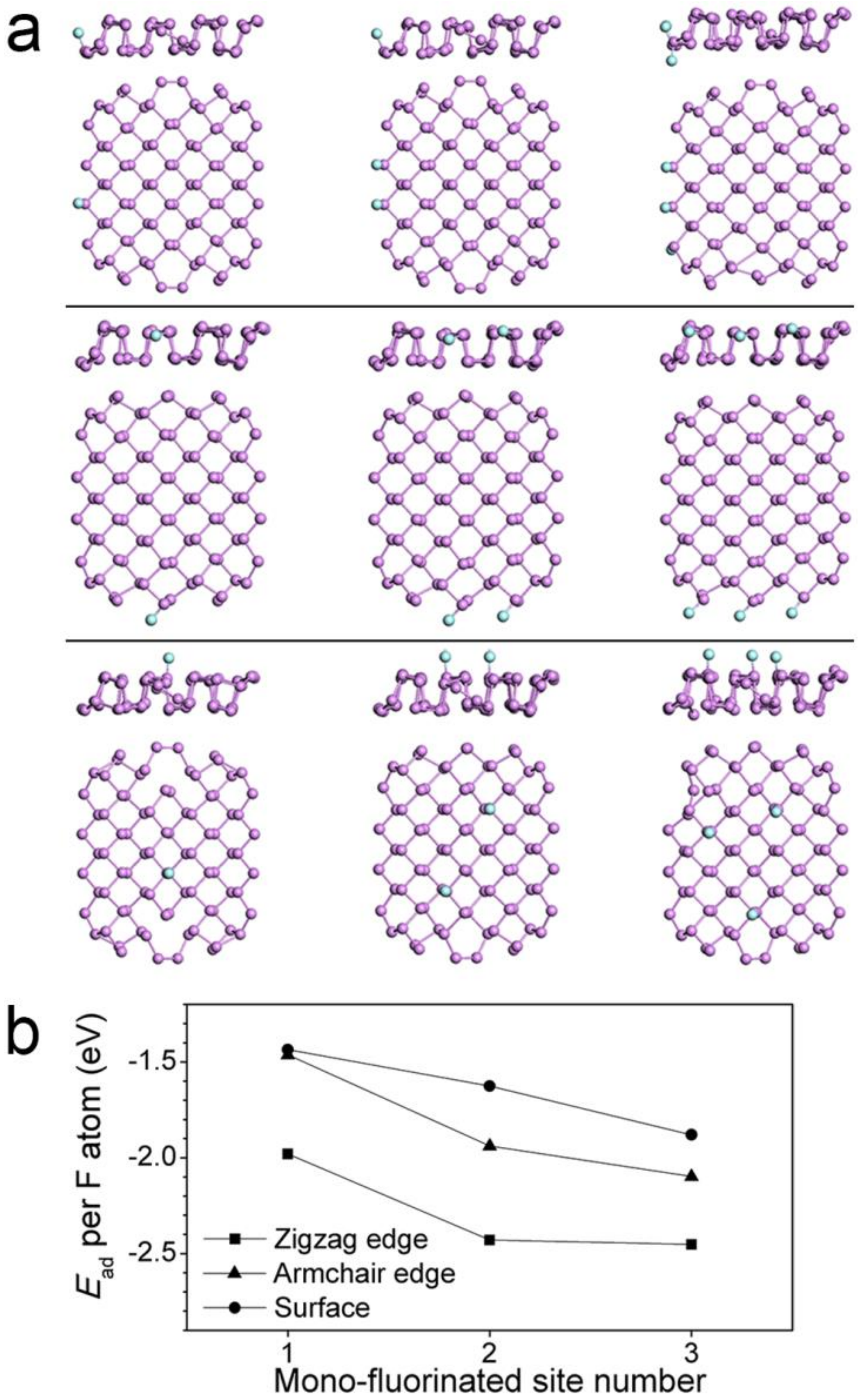

Figure S4. (a) Side and top views of optimized F-BPQD structures with 1–3 mono-fluorination sites in zigzag edge, armchair edge, or surface. (b) Variation of E_{ad} per F adatom with the number of mono-fluorination site.

The adsorption characteristics of F adatoms on F-BPQDs were investigated by DFT. The optimized F-BPQD models with different mono-fluorination sites are shown in Figure S4a. The F-BPQDs with 1–3 mono-fluorination sites in the zigzag edge, armchair edge, or surface are all thermodynamically stable, but structural distortions can be observed for these F-BPQDs. The calculated adsorption energies (E_{ad}s) per F adatom for the mono-fluorination sites are given in Figure S4b. It can be seen that, generally, F adatom in the zigzag edge mono-fluorination site has a smaller (or more negative) E_{ad} than that in the armchair edge and surface mono-fluorination sites, suggesting that the zigzag edge of BPQDs is more favorable to fluorination. It is also shown that E_{ad} per F atom decreases with the increase of the number of mono-fluorination sites for all three cases, indicating that the stability of the system increases as mono-fluorination site number increases. The bi- and tri-fluorination sites of F-BPQDs were also calculated to be stable, except for the surface tri-fluorination site (the corresponding optimized structures and E_{ad}s are shown in Figure S5 in the following page). E_{ad} per F atom in both bi- and tri-fluorination sites is larger than that in mono-fluorination site, and the zigzag edge bi- and tri-fluorination sites also have a smaller E_{ad} than the armchair edge and surface ones.

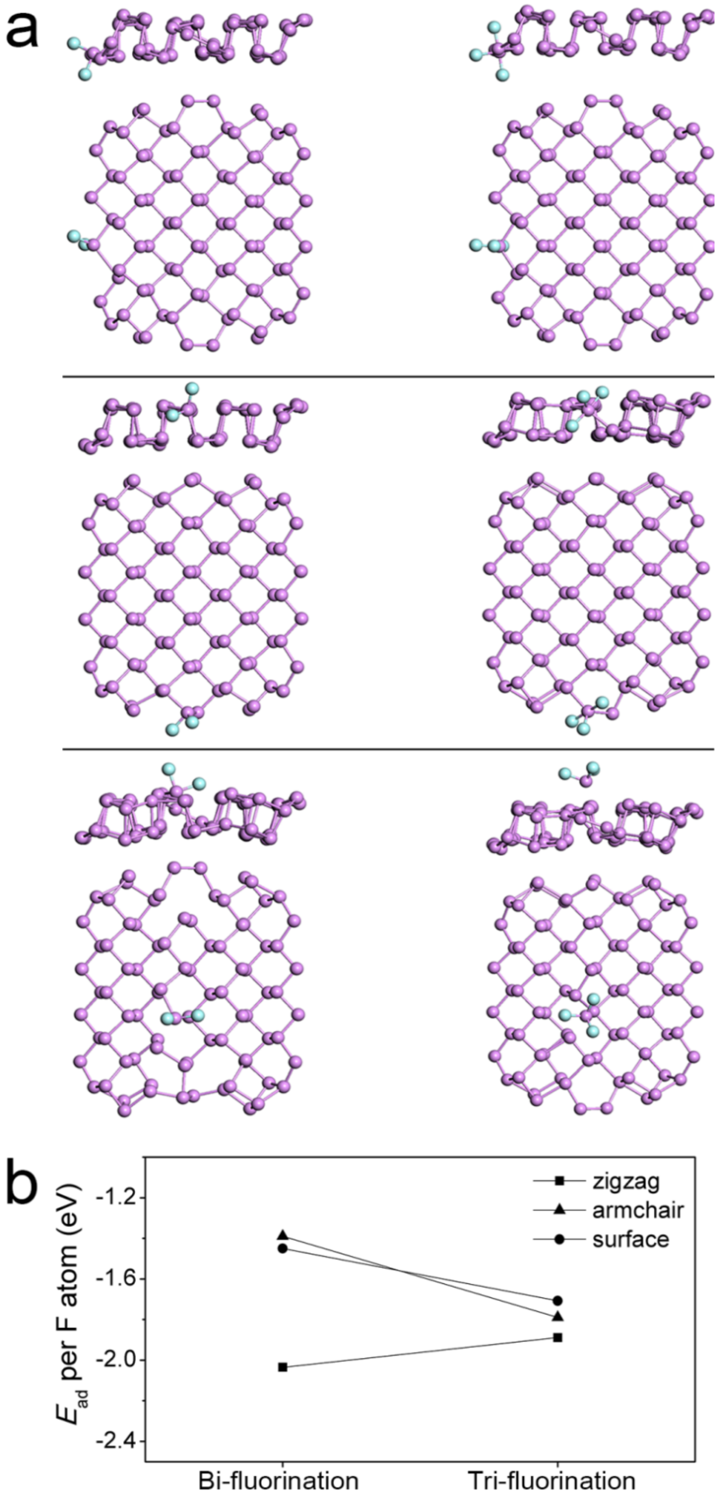

Figure S5. (a) Side and top views of optimized F-BPQD structures with a bi- or tri-fluorination site in zigzag edge, armchair edge, or surface. (b) Variation of E_{ad} per F adatom with fluorination site type. It is worth noting that the surface tri-fluorination site was relaxed to decomposed from the F-BPQD structure, and thus was actually impossible. E_{ad} of the F adatom in surface tri-fluorination site given in (b) is nominal and is for comparison.

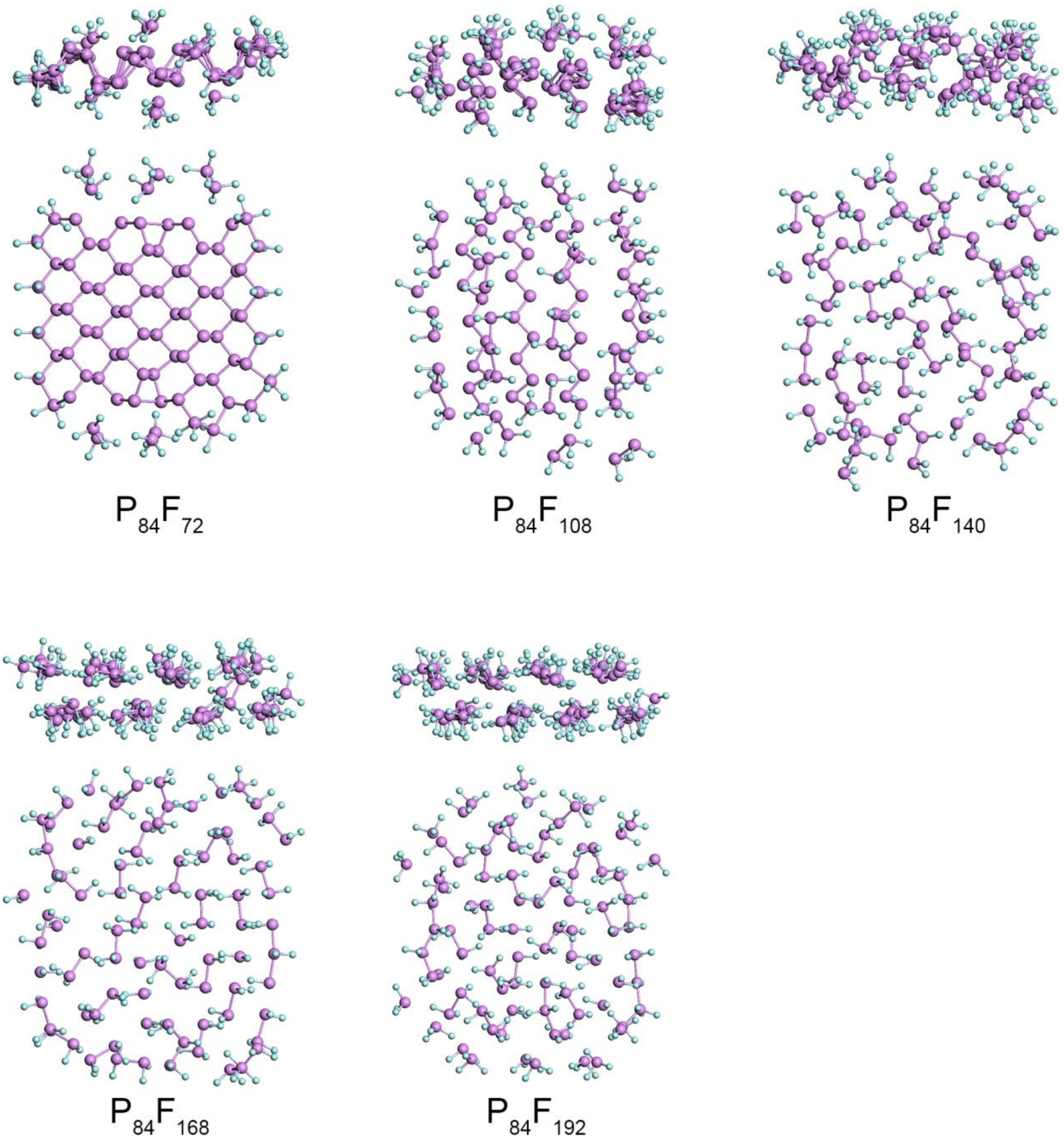

Figure S6. Optimized atomic structures of nominal F-BPQDs $P_{84}F_{72}$, $P_{84}F_{108}$, $P_{84}F_{140}$, $P_{84}F_{168}$, and $P_{84}F_{192}$, with D_F = 0.86, 1.29, 1.67, 2.0, and 2.29, respectively. $P_{84}F_{72}$ was with fully tri-fluorinated edge sites. $P_{84}F_{108}$ was with fully bi-fluorinated edge and single surface sites. $P_{84}F_{140}$, $P_{84}F_{168}$, and $P_{84}F_{192}$ have fully bi-fluorinated edge and double surfaces sites. $P_{84}F_{192}$ was bond-saturated. All these assumed structures had a D_F beyond the fluorination limit of 0.76, and thus were energetically unfavorable. The decomposition degree increased as D_F increased.

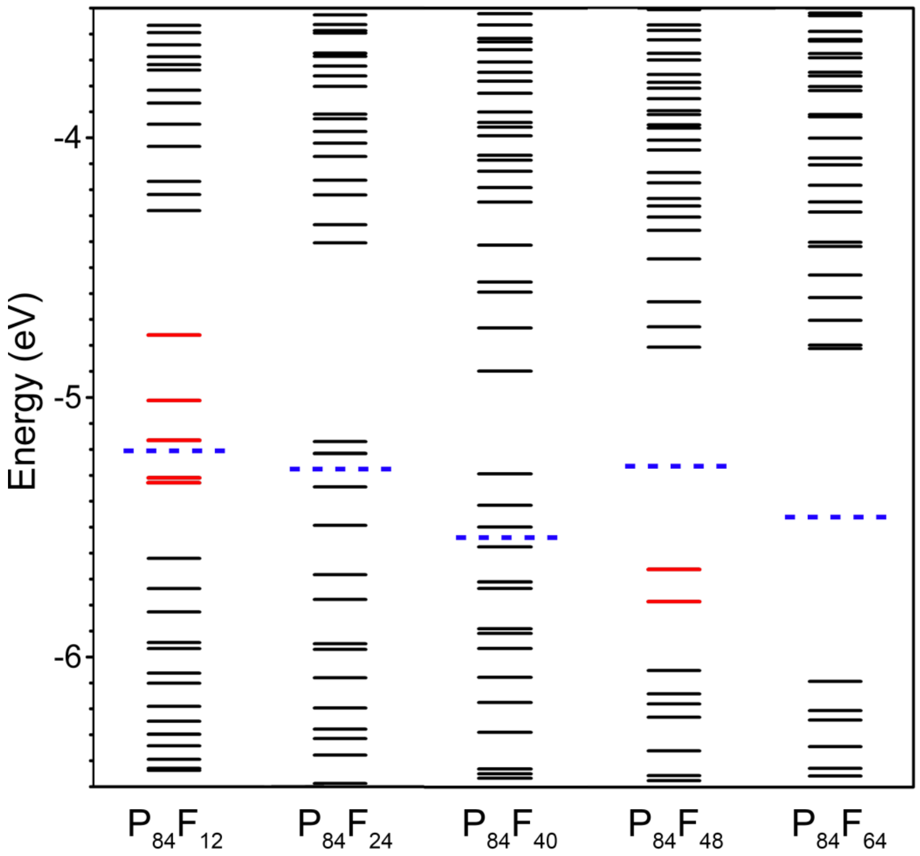

Figure S7. Energy levels of $P_{84}F_{12}$, $P_{84}F_{24}$, $P_{84}F_{40}$, $P_{84}F_{48}$, and $P_{84}F_{64}$ near the Fermi level. The red solid lines denote the IGS levels existed in the $P_{84}F_{12}$ and $P_{84}F_{48}$ F-BPQD with edge P atoms having CN ≠ 3 or 5. The dashed blue lines denote the Fermi levels of each F-BPQD. The two energy levels nearest to the bottom and the top of the Fermi level are the HOMO and LUMO levels for each BPQD, respectively.

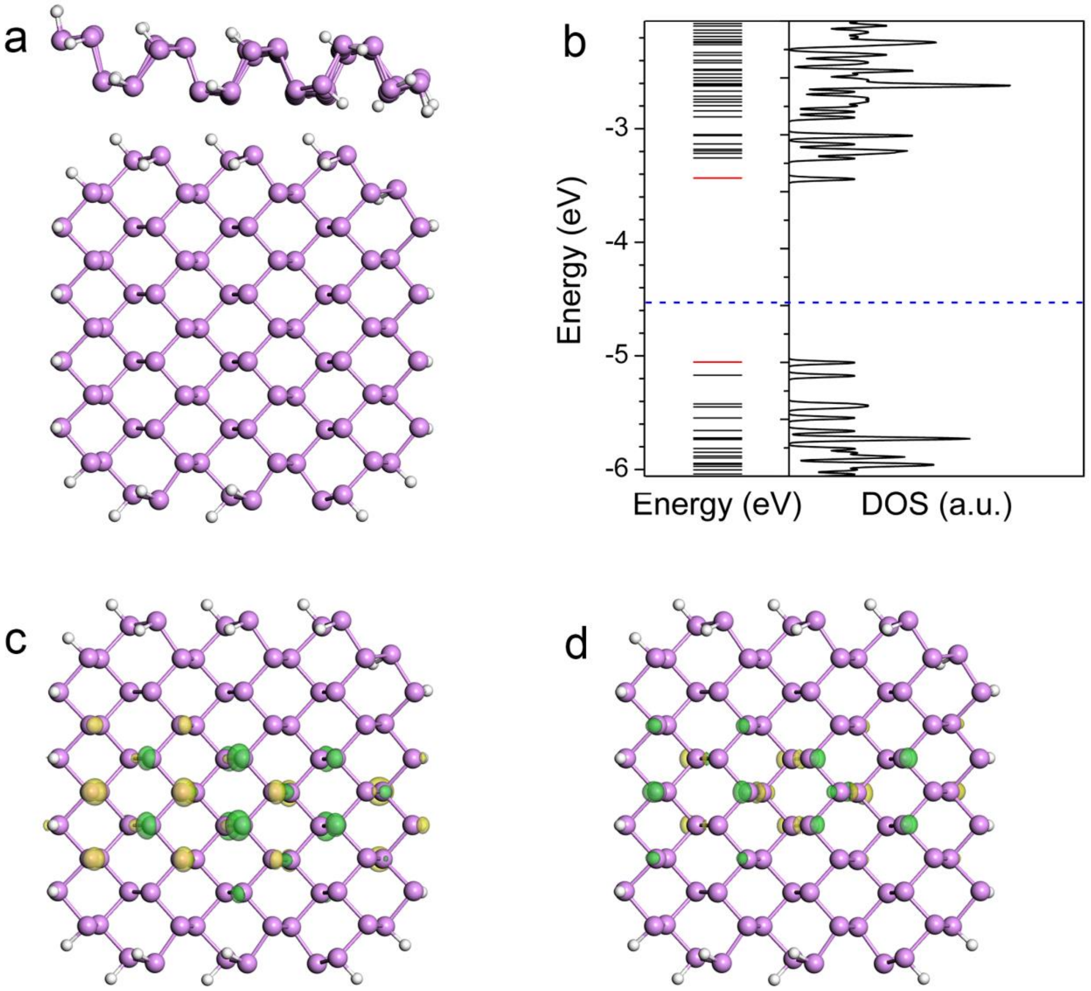

Figure S8. (a) Side and top view of optimized H-passivated BPQD $P_{84}H_{24}$. (b) Energy spectrum and DOS of $P_{84}H_{24}$. The red solid lines denote the HOMO and LUMO energy levels. The dashed blue line denotes the Fermi level. Wave-function iso-surfaces of (c) HOMO and (d) LUMO of $P_{84}H_{24}$. These results were calculated by using the PBE exchange-correlation functional at the GGA level, which are in good agreement with previous results calculated by using the B3LYP exchange-correlation functional (Ref. X. Niu, H. Shu, Y. Li, J. Wang, *J. Phys. Chem. Lett.* **2017**, *8*, 161).
